# In Vitro Antibacterial Activity of Commercial Essential Oils Against *Lactococcus garvieae*

**DOI:** 10.3390/microorganisms14091883

**Published:** 2026-08-25

**Authors:** Faik Sertel Secer

**Affiliations:** 1 Faculty of Agriculture, Department of Fisheries and Aquaculture, Ankara University, 06110 Ankara, Turkey; sertelsecer@yahoo.com; 2 Aquaterra Co., Ltd., Ankara University Technopolis, 06830 Ankara, Turkey

**Keywords:** lactococcosis, aquaculture, antibiotic resistance, broth microdilution, essential oils, *Lactococcus garvieae*, rainbow trout

## Abstract

Lactococcosis, caused by *Lactococcus garvieae*, is a persistent problem of farmed rainbow trout, and the reduced efficacy of conventional antibiotics has prompted interest in plant-derived alternatives. The in vitro antibacterial activity of five commercial essential oils from *Melaleuca alternifolia*, *Mentha piperita*, *Citrus limon*, *Rosmarinus officinalis* and *Lavandula hybrida* was evaluated against six *L. garvieae* isolates from rainbow trout. Minimum inhibitory and minimum bactericidal concentrations (MIC and MBC) were determined by broth microdilution, and the mode of action was defined by the MBC/MIC ratio; susceptibility to oxytetracycline (OTC) and trimethoprim–sulfamethoxazole (SXT) was also assessed. The major constituents were terpinen-4-ol (tea tree), menthol (peppermint), limonene (lemon), 1,8-cineole (rosemary) and linalool (lavandin). Activities were strain-dependent, with MIC values mostly from 6.25 to 100% (*v*/*v*) (single lowest, 1.5625% for rosemary), and lemon was among the least active, but the Kruskal–Wallis test showed no significant differences among oils (*p* > 0.05), so these differences are descriptive only. Where effective, the oils acted mainly through a bactericidal mechanism (MBC/MIC ≤ 4), but only at high concentrations, so their practical significance appears limited. All six isolates were resistant to OTC (MIC 8–16 µg/mL) and SXT (MIC 8–32 µg/mL). In vivo studies are required before practical use.

## 1. Introduction

Bacterial disease is frequently encountered in the aquaculture industry and has been associated with high mortality and considerable economic losses [1]. The Gram-positive bacterium *Lactococcus garvieae*, the causative agent of lactococcosis, is responsible for a prevalent infection in rainbow trout farms and may compromise the sustainability of intensive production [1,2,3]. Lactococcosis typically presents as a hyperacute haemorrhagic septicaemia, with outbreaks occurring mainly when water temperatures rise above 15–16 °C during the summer months [4,5]. The economic consequences of these outbreaks are considerable. In rainbow trout farms, cumulative mortality during natural outbreaks commonly reaches 40–50% of stock [5], and experimental challenges have produced cumulative mortalities of up to 80–90% at permissive temperatures [6]. Because the disease preferentially affects fish approaching market size, the economic damage per lost fish is disproportionately high; beyond direct mortality, losses also arise from reduced growth rates, treatment costs, downgrading at harvest and increased susceptibility to secondary infections [7]. Although disease-specific estimates are scarce, infectious diseases as a whole are estimated to cost global aquaculture approximately USD 6 billion annually [8], and the annual loss attributable to lactococcosis outbreaks in Iranian rainbow trout farming alone has been estimated at about USD 23 million [9]. The disease is endemic in European and Asian aquaculture, including Turkiye, one of the world’s leading producers of farmed rainbow trout, and is now expanding into the Americas [10]. Moreover, because outbreak severity is strongly temperature-dependent, with experimental mortalities rising from 0–7% at 13 °C to 59–91% at 18 °C [6], the prevalence of lactococcosis may therefore be favoured by progressive water warming. These recurrent and costly outbreaks, combined with the declining efficacy of licensed antibiotics, underline the need for alternative antimicrobial strategies. However, the conventional antibiotics used to treat such infections have been associated with the emergence of drug-resistant strains, reduced therapeutic efficacy and environmental accumulation [11,12]. Accordingly, alternative therapeutic strategies against bacterial pathogens have received increasing attention in recent studies.

Essential oils (EOs) obtained from various plant materials have been investigated as potential antibacterial agents for use in aquaculture [13,14,15,16,17]. EOs have been reported to show antibacterial activity against a range of bacterial species, with possible applications in food safety and medicine [18,19]. In vitro activity has also been reported against Gram-positive and Gram-negative bacteria isolated from infected fish [20,21,22,23,24]. In aquaculture, EOs have been examined both as direct antibacterial agents and as dietary supplements [21,22,23,25,26,27,28]. Their multitarget action is thought to lower the likelihood of single-mechanism resistance [18,27].

The interest in EOs is partly related to their natural origin, general biodegradability, broad antibacterial spectrum and multitarget modes of action [16,17,20,24]. However, their application in aquaculture is constrained by several factors, including variability in chemical composition among batches and suppliers, limited water solubility, volatility, possible effects on the sensory characteristics of fish products, and the need to establish species-specific effective doses. Moreover, the reported antibacterial activity varies considerably between studies, and many EOs are effective only at relatively high concentrations, which may limit their practical use [20,29,30]. Accordingly, the evaluation of commercial EOs under standardized conditions has been regarded as important before they are proposed for fish health management [14,15,27,31].

The minimum inhibitory concentration (MIC) and minimum bactericidal concentration (MBC) assays provide useful initial measures of the antibacterial activity of EOs before in vivo evaluation. The MIC is defined as the lowest concentration that inhibits visible bacterial growth, whereas the MBC is the lowest concentration required to reduce the initial inoculum by 99.9%. When evaluated together, these values can help distinguish inhibitory from bactericidal effects [32,33,34,35]. However, to date, the activity of EOs against *L. garvieae* has been examined in only a limited number of studies [36,37,38]. Therefore, this study was carried out to evaluate the in vitro antibacterial activity of five commercially available essential oils, obtained from tea tree (*Melaleuca alternifolia*), peppermint (*Mentha piperita*), lemon (*Citrus limon*), rosemary (*Rosmarinus officinalis*) and lavandin (*Lavandula hybrida*), against the fish pathogen *L. garvieae*. These oils were selected to represent different botanical origins and chemical profiles and were compared under the in vitro conditions. To date, no standardized side-by-side comparison of the MIC and MBC values of these five commercial essential oils against *L. garvieae* field isolates has been reported in conjunction with the antibiotic susceptibility profiles of the same isolates and full disclosure of replicate-level data. The present study addresses this gap.

## 2. Materials and Methods

### 2.1. Bacterial Strains

Strains of *L. garvieae* previously isolated from moribund or dead farmed rainbow trout were used in the in vitro antimicrobial susceptibility tests. The identifications of isolates were established based on morphological, cultural, and biochemical tests according to Buller [39], as well as MALDI-TOF (Biotyper 3.0; Microflex LT; Bruker Daltonics GmbH, Bremen, Germany; Maldi Biotyper Real-Time Classification (RTC) version.12-13.532 MSP) and VITEK 2 (bioMérieux, Marcy l’Etoile, France) rapid tests. The isolates, maintained in the author’s culture collection until testing, were subcultured twice on fresh medium prior to testing, and their purity and identity reconfirmed. The bacteria were passaged on Mueller–Hinton agar (MHA) (Merck, Cat No. 1.05437.0500) plates and subsequently transferred to Mueller–Hinton broth (MHB) (Condalab, Cat.1214.00); cultures were prepared by incubation under aerobic conditions at 22–24 °C, reflecting typical rearing temperatures, for 24 h. The bacterial pathogens and their origins used in the study are given in Table 1.

### 2.2. Essential Oils

In this study, five EOs of tea tree (*Melaleuca alternifolia* L.), peppermint (*Mentha piperita* L.), lemon (*Citrus limon* L.), rosemary (*Rosmarinus officinalis* L.), and lavandin (*Lavandula hybrida* L.) were obtained from Botalife, Turkiye (Table 2).

### 2.3. Inoculum Preparation and Antibacterial Assay

The MIC values were determined using the broth microdilution method in 96-well plates. Bacterial strains were spread on Mueller–Hinton Agar (MHA) plates. After overnight incubation, bacterial cultures (~1.1 × 10^8^ CFU/mL) were transferred to Mueller–Hinton Broth (MHB) and adjusted to McFarland 0.5 with an optical density (OD_600_) of absorbance ~0.1 [40]. This suspension was then diluted in MHB so that, after addition to the wells, the final inoculum was approximately ~5 × 10^5^ CFU/mL per well.

Due to the lipophilic nature of EOs, they were processed to achieve a homogeneous mixture with a water-based bacterial suspension by employing Tween 20. A stock of each EO was prepared in MHB containing 1% Tween 20, and two-fold serial dilutions were performed so that the final concentrations in the wells ranged from 0.098 to 100% (*v*/*v*), corresponding to the neat oil as 100%.

Antibacterial activity was assessed using the microdilution method in accordance with the guidelines of the Clinical and Laboratory Standards Institute (CLSI) [41], and antibiotic susceptibility testing was performed with oxytetracycline (OTC) and trimethoprim–sulfamethoxazole (SXT) following the protocols of the CLSI, the European Committee on Antimicrobial Susceptibility Testing (EUCAST) [42] and González-Martín et al. [43].

The antibiotics were dissolved in sterile distilled water. The OTC and SXT were evaluated at concentrations ranging from 0.13 to 256 µg/mL. A quality-control reference strain was not included; accordingly, the antibiotic results were used only to document the resistance status of the isolates and not to establish an absolute potency scale.

The MIC assay was performed using a microdilution method. A 100 µL volume of MHB was dispensed into all wells. A 100 µL aliquot of the highest EO concentration was then added to the first well and serially two-fold diluted along the row, discarding 100 µL from the last well so that each well retained 100 µL. Finally, 100 µL of bacterial inoculum (McFarland 0.5) was added to every well, giving a final volume of 200 µL and a further two-fold dilution of each EO concentration. The plates were incubated at 22–24 °C for 24 h, and the MIC was defined as the lowest concentration in the first well without visible bacterial growth [44]. Incubation at 22–24 °C in MHB was chosen to reflect the rearing temperature and growth requirements of this fish pathogen; this deviates from standard CLSI incubation conditions.

A 200 µL volume of MHB was used as a negative control to verify the absence of contamination. In contrast, the growth control was prepared by mixing 100 µL of pure culture medium (MHB) with 100 µL of bacterial suspension. A solvent control containing 1% Tween 20 without essential oil was included. All tests were repeated three times.

A 5 µL sample from the 96-well MIC plate containing the bacteria, EO and MHB mixture was subcultured onto MHA plates using a 48-pin Multi-Blot Replicator. The plates were incubated at 22–24 °C for 24 h, and the MBC was defined as the lowest concentration showing complete absence of visible colonies on the agar plate [45,46]. As for the MIC, all MBC assays were performed in independent triplicate for each EO–strain combination.

The MBC/MIC ratio was used to ascertain the nature of the agent’s activity, distinguishing between bacteriostatic and bactericidal effects. Specifically, a ratio of ≤4 signified bactericidal activity, whereas a ratio exceeding 4 indicated bacteriostatic activity [33].

### 2.4. Statistical Analysis

MIC, MBC and MBC/MIC values were determined from three independent replicates for each oil–isolate combination and were reported as the modal value per isolate (*n* = 6 isolates per essential oil). Because the data were ordinal (two-fold serial dilutions) and not normally distributed, differences among the five essential oils were evaluated with the non-parametric Kruskal–Wallis H test, with the essential oil as the grouping factor. Values recorded as >100% (ND) were treated as missing. Results are expressed as median (minimum–maximum). A probability level of *p* < 0.05 was considered statistically significant. All analyses were performed in IBM SPSS Statistics (v. 26).

## 3. Results

### 3.1. Analysis of EOs

The main constituents of the examined EOs were determined by GC-MS (Table 3). In tea tree (*M. alternifolia*) oil, terpinen-4-ol was the major component (22.37%), followed by γ-terpinene (16.61%), 2-carene (11.92%), p-cymene (8.36%), α-pinene (6.03%), 1,8-cineole (5.89%) and terpinolene (5.79%). In peppermint (*M. piperita*) oil, menthol was the most abundant component (29.07%), followed by menthone (25.61%) and a menthone isomer (14.76%), with limonene also present (5.43%). In lemon (*C. limon*) oil, limonene was the dominant component (40.52%), followed by 3-carene (14.05%), γ-terpinene (11.15%), p-cymene (6.74%) and α-pinene (5.71%). In rosemary (*R. officinalis*) oil, 1,8-cineole was the major component (30.06%), followed by α-pinene (11.51%), camphor (10.69%), β-pinene (7.99%), camphene (7.03%), limonene (6.45%) and o-cymene (5.03%). In lavandin (*L. hybrida*) oil, linalool (26.19%), linalyl acetate (17.47%), camphor (9.16%) and 1,8-cineole (9.13%) were the main constituents.

### 3.2. Biochemical Characterization of Isolates

All six *L. garvieae* isolates previously identified were reconfirmed by morphological, cultural and biochemical testing according to Buller [39]. No biochemical differences were observed among the isolates S1–S6; their phenotypic characteristics were identical and consistent with *L. garvieae*.

### 3.3. Findings of Antibacterial Analysis of the EOs

For the tea tree oil, the MIC values ranged from 3.125 to 50%, with S1 the most susceptible and S2 and S6 the least; the MBC values ranged from 12.5 to 100%, and the MBC did not exceed the MIC by more than fourfold, so tea tree oil was classified as bactericidal against all six isolates (Table 4). With peppermint oil, S4 and S5 were inhibited at 6.25% and the remaining strains at 12.5 to 50%; the MBC values ranged from 25 to 100%, and MBC/MIC ratios of 2 to 4 placed all six strains in the bactericidal group (Table 5). In the case of lemon oil, the MIC values ranged from 25 to 100%, the highest recorded for S3, and the MBC values from 50 to 100%; none of the ratios exceeded four, so every strain was bactericidal (Table 6). With respect to rosemary oil, S1 was inhibited at 1.5625%, whereas S5 and S6 showed MIC values of 100% (Table 7). For S5 and S6, no MBC was reached within the concentration range tested, so neither a ratio nor a classification was assigned. For lavandin oil, the MIC values ranged from 6.25 to 50% and the MBC values from 12.5 to 100%; the ratios remained at or below four, and all six strains were bactericidal (Table 8). The individual replicate MIC and MBC values (R1–R3) underlying the modal results summarised in Table 4, Table 5, Table 6, Table 7 and Table 8 are presented in full in Table A1. No growth occurred in the negative-control wells containing MHB alone, confirming the absence of contamination, whereas the growth-control wells (MHB inoculated with the bacterial suspension) showed visible growth. The solvent-control wells, containing 1% Tween 20 without essential oil, showed no inhibition of bacterial growth.

### 3.4. Antibiotic Susceptibility

The OTC and SXT susceptibility results are shown in Table 9 and Table A2. OTC MIC values were 8 or 16 µg/mL and SXT MIC values ranged from 8 to 32 µg/mL. All six strains were categorised as resistant to both antibiotics.

### 3.5. Statistical Results

The MIC, MBC and MBC/MIC values were compared among the five oils with the Kruskal–Wallis test. No significant differences were detected among the five oils for any parameter (MIC: H = 3.455, df = 4, *p* = 0.485; MBC: H = 6.662, df = 4, *p* = 0.155; MBC/MIC: H = 4.041, df = 4, *p* = 0.400; Table 10). Accordingly, the observed ranking of the oils should be regarded as descriptive only.

## 4. Discussion

In this study, the in vitro antibacterial activity of five commercial EOs was assessed against six field isolates of *L. garvieae* from rainbow trout. The oils differed in their major volatile constituents, and their antibacterial performance varied numerically between isolates; however, the differences between oils were not statistically supported.

On a descriptive basis, rosemary produced the single lowest MIC value against any individual strain, whereas on a median basis it did not rank above tea tree, peppermint or lavandin, and lemon was among the least active. This pattern is broadly consistent with earlier reports that the antibacterial activity of EOs is oil-specific and depends on both the plant source and the target organism [13,15,20]. However, when the values were compared with the Kruskal–Wallis test, no significant differences were detected among the oils (Kruskal–Wallis, all *p* > 0.05). Accordingly, the observed ranking reflects descriptive trends and strain-level differences rather than a statistically supported hierarchy of potency. This finding is relevant because the reported activity of EOs against *L. garvieae* has been variable across studies, and part of this variability may arise from differences in oil composition, isolate origin and testing methodology [36,37,38].

The GC-MS analyses provide a basis for relating activity to composition. Tea tree oil was dominated by terpinen-4-ol, which is generally regarded as the principal contributor to the antibacterial activity of M. alternifolia oil and is thought to act on the cytoplasmic membrane [48]. Rosemary oil was rich in 1,8-cineole and camphor, and lavandin oil in linalool and linalyl acetate, which are oxygenated terpenoids frequently associated with antibacterial effects [13,49]. By contrast, lemon oil was dominated by limonene, a hydrocarbon monoterpene with comparatively limited antibacterial activity, which is consistent with the weak effect observed in this study [13]. These associations are consistent with the view that the activity of an EO reflects its dominant oxygenated constituents rather than its total terpene content. However, as the differences among the oils were not statistically significant, these composition–activity relationships should be regarded as tentative associations rather than demonstrated causal effects [50,51].

Notably, the effective concentrations recorded (MIC mostly 6.25 to 100%) were several orders of magnitude higher than the values reported for other plant-derived antibacterials against *L. garvieae*, which typically fall in the µg/mL range; for example, Fereidouni et al. [36] reported MIC values of 105–510 µg/mL for the most active extracts and oils [9]. On a mass basis the oils tested in this study appeared markedly less active than those ethanolic extracts, which is unexpected given that essential oils are usually more potent than crude extracts. A difference of this magnitude is unlikely to be explained by oil composition alone and may partly reflects the way concentrations were prepared and expressed (*w*/*v* versus *v*/*v*) across studies; the present values should therefore be interpreted with caution and compared with earlier reports only after conversion to a common unit.

The MBC/MIC ratios suggest that, where the EOs were effective, they acted mainly through a bactericidal rather than a bacteriostatic mechanism, since all effective combinations had ratios of ≤4 [33,34,35].

From an applied perspective, the concentrations at which activity was recorded argue strongly against the direct therapeutic use of these oils in their present form. The observed MIC values lie in the gram-per-litre range and thus exceed fish-tolerated concentrations by three to four orders of magnitude; in rainbow trout, the 96-h LC50 values of thyme essential oil and its major constituent thymol are only 6.6 and 2.6 mg/L, respectively [52].

Consequently, any practical application of these oils would require strategies that decouple antibacterial efficacy from host exposure, such as dietary administration at sublethal doses, encapsulation, or the use of purified constituents as lead structures for further development rather than as ready-to-use therapeutics. Delivery through feed is similarly constrained. The inclusion levels that would be required are difficult to reconcile with feed palatability and intake, with oil volatility during feed manufacture, and with product cost and possible sensory effects on the final product [53]. Moreover, the benefits reported for dietary EOs in fish have generally been obtained at low, immunomodulatory doses rather than at directly antibacterial ones [9]. A further limitation concerns the selection of a candidate oil. Because no significant differences were detected among the five oils, the present data do not provide an evidence-based criterion for preferring any single oil for further development, and such a choice would have to rely on descriptive trends and composition-based expectations rather than on demonstrated superiority. Taken together, these considerations suggest that the tested commercial oils are unlikely to be suitable as stand-alone therapeutics for lactococcosis, and that their potential value should instead be sought in optimized formulations and combination strategies. Nanoencapsulation and nanoemulsification can mitigate the low water solubility, volatility and host toxicity of EOs while preserving or enhancing their antibacterial activity [54,55]. In addition, checkerboard studies have shown that EOs may act synergistically with oxytetracycline and can reduce the effective antibiotic concentration against multidrug-resistant bacteria by up to three orders of magnitude [56]. Given that all six isolates examined in the present study were categorised as resistant to OTC and SXT, EO–antibiotic combinations delivered through encapsulated systems represent a promising route by which the modest activity observed could be translated into practical benefit, and they provide a testable hypothesis for future in vivo studies.

All six isolates were categorised as resistant to both OTC and SXT, with modal MIC values of 8–16 µg/mL for OTC and 8–32 µg/mL for SXT. The reduced susceptibility to SXT is consistent with previous reports. Öztürk et al. [47] could not establish an epidemiological cutoff value for SXT in *L. garvieae* because the MIC distribution fell largely outside the tested range, and comparable elevated values were recorded by González-Martín et al. [43]. The OTC MICs were also comparatively high and, against the provisional cutoff of Öztürk et al. [47], would classify all six isolates as non-wild type, in contrast to the low MICs described for *L. garvieae* from Mediterranean marine fish [57,58]. Several limitations nonetheless constrain the interpretation of these MIC values. Neither CLSI nor EUCAST provides interpretive breakpoints specific to *Lactococcus* from aquatic animals, so the general criteria applied in this study were indicative rather than definitive.

The present study was confined to MIC and MBC endpoints. Practical application of EOs in aquaculture would require a wide range of further studies. In particular, the effects of these oils on bacterial membrane integrity, their capacity to inhibit and eradicate established biofilms, and their impact on the expression of virulence- and resistance-associated genes assessed by qPCR should be examined. Such mechanistic and applied data would clarify whether the in vitro activity observed here can be translated into safe and effective control strategies against *L. garvieae* in aquaculture.

## 5. Conclusions

Under the conditions of this study, all six field isolates of *L. garvieae* were resistant to both OTC and SXT, based on the general and provisional criteria available for this species. This underscores the need for alternative approaches. Against this background, the five commercial essential oils showed measurable but statistically indistinguishable in vitro activity, and their apparent ranking should only be treated as descriptive. Where effective, the oils acted mainly through a bactericidal mechanism (MBC/MIC ≤ 4), but only at high concentrations, so their practical significance is limited. Based on these results, the tested EOs should be regarded as candidates for optimization or combination approaches, such as nanoencapsulation to improve their solubility and stability or combination with conventional antibiotics to screen for possible synergy, rather than as stand-alone treatments. However, further studies on in vivo efficacy and toxicity, together with standardisation of oil composition, are necessary before practical application in aquaculture can be considered.

## Figures and Tables

**Table 1:** Bacterial pathogens isolated from rainbow trout.

No	Code	Strain	Source
1	S1	3-Lg-S	Spleen
2	S2	5-Lg-S	Spleen
3	S3	8-Lg-S	Spleen
4	S4	12Lg-L	Liver
5	S5	14Lg-L	Liver
6	S6	18Lg-K	Kidney

**Table 2:** Names, botanical origin and batch details of the EOs.

Essential Oil	Plant Origin	Lot Number
Tea Tree EO	*Melaleuca alternifolia*	P.NO: 28.50111
Peppermint EO	*Mentha piperita*	P.NO: 50.11
Lemon EO	*Citrus limon*	P.NO: 77.50310
Rosemary EO	*Rosmarinus officinalis*	P.NO: 195.2211
Lavandin EO	*Lavandula hybrida*	P.NO: 75.50110

**Table 3:** Major chemical composition of the EOs (>5%).

Essential Oil	Compound	CAS Number	RT (min)	Conc. (%)
Tea tree (*Melaleuca alternifolia*)	α-Pinene	7785-70-8	10.060	6.03
	2-Carene	554-61-0	12.420	11.92
	γ-Terpinene	99-85-4	13.480	16.61
	Terpinolene	586-62-9	14.120	5.79
	Eucalyptol (1,8-cineole)	470-82-6	14.370	5.89
	p-Cymene	99-87-6	15.160	8.36
	Terpinen-4-ol	562-74-3	26.080	22.37
Peppermint (*Mentha piperita*)	Limonene	5989-27-5	12.491	5.43
	Menthone	14073-97-3	24.750	25.61
	Menthone	89-80-5	26.120	14.76
	Menthol	89-78-1	26.611	29.07
Lemon (*Citrus limon*)	α-Pinene	7785-70-8	10.064	5.71
	3-Carene	13466-78-9	11.400	14.05
	Limonene	5989-27-5	12.626	40.52
	γ-Terpinene	99-85-4	13.472	11.15
	p-Cymene	99-87-6	15.168	6.74
Rosemary (*Rosmarinus officinalis*)	α-Pinene	7785-70-8	10.068	11.51
	Camphene	5794-04-7	10.825	7.03
	β-Pinene	127-91-3	11.383	7.99
	Limonene	5989-27-5	12.500	6.45
	Eucalyptol (1,8-cineole)	470-82-6	14.441	30.06
	o-Cymene	527-84-4	15.155	5.03
	Camphor	464-49-3	27.469	10.69
Lavandin (*Lavandula hybrida*)	Eucalyptol (1,8-cineole)	470-82-6	14.360	9.13
	Linalool	78-70-6	22.657	26.19
	Linalyl acetate	115-95-7	23.198	17.47
	Camphor	464-49-3	27.461	9.16

**Table 4:** MIC, MBC and bacteriostatic/bactericidal classification of tea tree (*Melaleuca alternifolia*) *.

	Strain	MIC (%)	MBC (%)	MBC/MIC	BS/BC
Tea Tree Oil	S1	3.125	12.5	4	BC
	S2	50	50	1	BC
	S3	25	25	1	BC
	S4	6.25	25	4	BC
	S5	6.25	25	4	BC
	S6	50	100	2	BC

* The reported MIC and MBC values strictly represent the modal value obtained from three independent replicate assays. Any observed biological variation between replicates was consistently confined to no more than one dilution step. BS, bacteriostatic; BC, bactericidal (BC if MBC/MIC ≤ 4; BS if MBC/MIC > 4).

**Table 5:** MIC, MBC and bacteriostatic/bactericidal classification of peppermint (*Mentha piperita*) oil *.

	Strain	MIC (%)	MBC (%)	MBC/MIC	BS/BC
Peppermint Oil	S1	25	100	4	BC
	S2	50	100	2	BC
	S3	50	100	2	BC
	S4	6.25	25	4	BC
	S5	6.25	25	4	BC
	S6	12.5	50	4	BC

* The reported MIC and MBC values strictly represent the modal value obtained from three independent replicate assays. Any observed biological variation between replicates was consistently confined to no more than one dilution step. BS, bacteriostatic; BC, bactericidal (BC if MBC/MIC ≤ 4; BS if MBC/MIC > 4).

**Table 6:** MIC, MBC and bacteriostatic/bactericidal classification of lemon (*Citrus limon*) oil *.

	Strain	MIC (%)	MBC (%)	MBC/MIC	BS/BC
Lemon Oil	S1	25	100	4	BC
	S2	50	50	1	BC
	S3	100	100	1	BC
	S4	25	50	2	BC
	S5	25	100	4	BC
	S6	50	100	2	BC

* The reported MIC and MBC values strictly represent the modal value obtained from three independent replicate assays. Any observed biological variation between replicates was consistently confined to no more than one dilution step. BS, bacteriostatic; BC, bactericidal (BC if MBC/MIC ≤ 4; BS if MBC/MIC > 4).

**Table 7:** MIC, MBC and bacteriostatic/bactericidal classification of rosemary (*Rosmarinus officinalis*) oil *.

	Strain	MIC (%)	MBC (%)	MBC/MIC	BS/BC
Rosemary Oil	S1	1.5625	3.125	2	BC
	S2	25	50	2	BC
	S3	12.5	12.5	1	BC
	S4	50	100	2	BC
	S5	100	ND	ND	-
	S6	100	ND	ND	-

* The reported MIC and MBC values strictly represent the modal value obtained from three independent replicate assays. Any observed biological variation between replicates was consistently confined to no more than one dilution step. BS, bacteriostatic; BC, bactericidal (BC if MBC/MIC ≤ 4; BS if MBC/MIC > 4). ND: Not determined (>100%).

**Table 8:** MIC, MBC and bacteriostatic/bactericidal classification of lavandin (*Lavandula hybrida*) oil *.

	Strain	MIC (%)	MBC (%)	MBC/MIC	BS/BC
Lavandin Oil	S1	6.25	25	4	BC
	S2	50	50	1	BC
	S3	50	100	2	BC
	S4	12.5	12.5	1	BC
	S5	12.5	50	4	BC
	S6	25	50	2	BC

* The reported MIC and MBC values strictly represent the modal value obtained from three independent replicate assays. Any observed biological variation between replicates was consistently confined to no more than one dilution step. BS, bacteriostatic; BC, bactericidal (BC if MBC/MIC ≤ 4; BS if MBC/MIC > 4).

**Table 9:** Antimicrobial susceptibility profiles (MIC values) and resistance categorization of *L. garvieae* against OTC and SXT.

Strain	OTC		SXT	
	MIC (µg/mL)	S/I/R	MIC (µg/mL)	S/I/R
S1	16	R	8	R
S2	16	R	32	R
S3	16	R	32	R
S4	8	R	16	R
S5	8	R	8	R
S6	16	R	32	R

OTC: Oxytetracycline, SXT: trimethoprim–sulfamethoxazole. MIC values are expressed as the consistent modal value of triplicate assays. S/I/R categories were assigned according to Öztürk et al. [47]. As neither CLSI nor EUCAST provides interpretive breakpoints specific to *L. garvieae* from aquatic animals, the general criteria applied were indicative rather than definitive. The provisional cutoff of Öztürk et al. [47] was used for OTC.

**Table 10:** Descriptive statistics [median (minimum–maximum)] and Kruskal–Wallis comparison of MIC, MBC and MBC/MIC values of the five essential oils against *Lactococcus garvieae* isolates (*n* = 6).

Essential Oil	MIC (%)	MBC (%)	MBC/MIC
Tea tree	15.625 (3.125–50)	25 (12.5–100)	3 (1–4)
Peppermint	18.75 (6.25–50)	75 (25–100)	4 (2–4)
Lemon	37.5 (25–100)	100 (50–100)	2 (1–4)
Rosemary	37.5 (1.5625–100)	31.25 (3.125–100)	2 (1–2)
Lavandin	18.75 (6.25–50)	50 (12.5–100)	2 (1–4)
Chi-square (H)	3.455	6.662	4.041
df	4	4	4
*p* value	0.485	0.155	0.400

Kruskal–Wallis H test; grouping variable: essential oil. No significant difference was found among the five essential oils for any parameter (all *p* > 0.05). MBC values > 100% (ND) were treated as missing; MBC and MBC/MIC for rosemary are based on *n* = 4 isolates.

## Data Availability

The original contributions presented in this study are included in the article. Further inquiries can be directed to the corresponding author.

## Abbreviations

The following abbreviations are used in this manuscript:

MIC	Minimum inhibitory concentration
MBC	Minimum bactericidal concentration
EOs	Essential oils
EO	Essential oil
MHB	Mueller–Hinton Broth
MHA	Mueller–Hinton Agar
CFU	Colony Forming Units
S	Susceptible
I	Intermediate
R	Resistant

## Appendix A

Individual replicate values (R1–R3) and the corresponding modal value are reported for each essential oil and antibiotic against the six *Lactococcus garvieae* isolates (S1–S6). As no replicate deviated by more than one two-fold dilution step from the modal value, all determinations were accepted as valid.

**Table A1:** MIC and MBC of EOs against *L. garvieae* isolates (%).

	Isolate	MIC				MBC			
		R1	R2	R3	Modal	R1	R2	R3	Modal
Tea tree oil (*Melaleuca alternifolia*)	S1	6.25	3.125	3.125	**3.125**	25	12.5	12.5	**12.5**
	S2	50	50	50	**50**	50	100	50	**50**
	S3	25	25	25	**25**	25	25	25	**25**
	S4	6.25	6.25	6.25	**6.25**	25	25	25	**25**
	S5	6.25	6.25	12.5	**6.25**	25	50	25	**25**
	S6	50	50	50	**50**	100	>100	100	**100**
Peppermint oil (*Mentha piperita*)	S1	25	50	25	**25**	100	100	100	**100**
	S2	50	50	50	**50**	100	>100	100	**100**
	S3	50	50	50	**50**	>100	100	100	**100**
	S4	12.5	6.25	6.25	**6.25**	25	25	50	**25**
	S5	6.25	12.5	6.25	**6.25**	25	25	25	**25**
	S6	12.5	12.5	25	**12.5**	50	100	50	**50**
Lemon oil (*Citrus limon*)	S1	50	25	25	**25**	100	100	100	**100**
	S2	50	50	50	**50**	100	50	50	**50**
	S3	100	100	>100	**100**	100	100	>100	**100**
	S4	25	50	25	**25**	50	50	50	**50**
	S5	25	25	25	**25**	>100	100	100	**100**
	S6	100	50	50	**50**	100	100	100	**100**
Rosemary oil (*Rosmarinus officinalis*)	S1	1.5625	3.125	1.5625	**1.5625**	3.125	6.25	3.125	**3.125**
	S2	25	25	25	**25**	50	50	50	**50**
	S3	12.5	12.5	12.5	**12.5**	12.5	25	12.5	**12.5**
	S4	50	100	50	**50**	100	>100	100	**100**
	S5	100	>100	100	**100**	>100	>100	>100	**ND**
	S6	100	100	100	**100**	>100	>100	>100	**ND**
Lavandin oil (*Lavandula hybrida*)	S1	6.25	12.5	6.25	**6.25**	25	25	25	**25**
	S2	50	50	50	**50**	50	50	50	**50**
	S3	50	50	50	**50**	>100	100	100	**100**
	S4	25	12.5	12.5	**12.5**	12.5	12.5	12.5	**12.5**
	S5	12.5	12.5	12.5	**12.5**	50	50	50	**50**
	S6	25	25	25	**25**	50	50	50	**50**

MIC and MBC expressed in %; each value is the modal result of three independent replicates (R1–R3). The bold column is the modal value used in the main analysis. ND, not determined (no bactericidal endpoint within the tested range, >100%). Values reported as >100 indicate no endpoint at the highest concentration tested.

**Table A2:** MIC of OTC and SXT against *L. garvieae* isolates (µg/mL).

Isolate	OTC				SXT			
	R1	R2	R3	Modal	R1	R2	R3	Modal
S1	16	16	16	**16**	8	8	8	**8**
S2	16	16	16	**16**	32	32	32	**32**
S3	16	16	16	**16**	32	64	32	**32**
S4	8	16	8	**8**	16	16	16	**16**
S5	8	8	8	**8**	8	8	8	**8**
S6	16	16	16	**16**	32	32	32	**32**

MIC expressed in µg/mL; each value is the modal result of three independent replicates (R1–R3). The bold column indicates the modal value used in the main analysis. As neither CLSI nor EUCAST provides interpretive breakpoints specific to *L. garvieae* from aquatic animals, the general criteria applied were indicative rather than definitive; the provisional cutoff of Öztürk et al. [47] was used for OTC.
